# Neurotoxic Snakebite Presenting with Early Neck Pain and Muscle Weakness: A Case Report of a Diagnostic Pitfall

**DOI:** 10.5811/cpcem.47366

**Published:** 2026-02-25

**Authors:** T. Neithiya, Jayan Jayapalan Nair, Krishnadutt Chavali

**Affiliations:** * All India Institute of Medical Sciences, Department of Forensic Medicine and Toxicology, Raipur, India; † ESIC Medical College & Hospital, Department of Forensic Medicine and Toxicology, Naroda-Bapunagar, India

**Keywords:** neurotoxic envenomation, flaccid quadriparesis, forensic toxicology, krait bite, case report

## Abstract

**Introduction:**

Neurotoxic envenomation often presents with non-specific neurological symptoms and minimal local signs, which can delay appropriate diagnosis and treatment. This is the first reported case of a neurotoxic snakebite presenting with an atypical symptom of unilateral neck pain.

**Case Report:**

A 12-year-old girl was referred to our emergency centre with neck weakness progressing to quadriplegia, attributed to a fall while playing. A diagnosis of acute flaccid paralysis secondary to cervical trauma was made and treated at the first hospital; however, she developed respiratory distress and was transferred to our centre. Clinical examination and computed tomography ruled out cervical cord injury. A diagnosis of neurotoxic envenomation was considered, given our centre’s high snakebite burden and the symptom of descending flaccid paralysis. Despite initiating antivenom and supportive treatment, the patient died. As the death was sudden and unexplained, medicolegal autopsy was done. Meticulous examination revealed a suspicious mark over the right foot. Chemical analysis on a skin sample from the site tested positive for snake venom, confirming envenomation.

**Conclusion:**

This case highlights the diagnostic challenge posed by atypical presentations of neurotoxic snakebite, especially in the absence of a clear history. In endemic areas, flaccid paralysis should prompt clinical suspicion of snakebite. Early recognition and timely administration of antivenom are crucial to prevent fatal outcomes. This case also underscores the need for strengthening diagnostic tools and forensic confirmation to avoid missed or delayed diagnoses, which carry serious medicolegal and public health implications.

## INTRODUCTION

Snakebites are recognized by the World Health Organisation as a neglected tropical disease, with an estimated 5.4 million bites annually leading to over 80,000 deaths in rural Asia and Africa.^1^ India accounts for nearly half of global snakebite mortality, with neurotoxic envenomation by kraits and cobras (Elapidae) as the leading cause.^2^ Children are especially vulnerable due to their smaller body mass and delayed access to care because of incomplete history.^3^ The descending paralysis seen in elapid bites can mimic other conditions such as Guillain-Barré syndrome or cervical spinal cord injury, which can lead to misdiagnosis and delayed use of anti-snake venom.^5^–^7^ Neurotoxic snakebites usually present with minimal or absent local signs, complicating early clinical recognition.^4^ In fatal cases, lack of overt bite marks and non-specific autopsy findings complicate forensic confirmation.^8^

We present a case of a neurotoxic snakebite masquerading as cervical spine trauma in a child with ambiguous history and no clear bite evidence. To the best of our knowledge, this is the first reported case of a neurotoxic snakebite presenting with the atypical symptom of unilateral neck pain, highlighting the diagnostic dilemma it can pose in the absence of clear bite evidence.

## CASE REPORT

A 12-year-old girl was referred to our paediatric emergency department with right-sided neck pain for 12 hours, followed by altered sensorium for three hours. The pain reportedly began after she fell on her back and hit her neck while playing with friends. The pain restricted neck movement and was followed by tingling and weakness in all limbs. Initially seen by a local physician, she was sent home on medications. Later, due to worsening weakness and confusion, she was taken to a hospital, where cervical trauma was suspected.

No history of seizures, fever, bleeding, abdominal pain, vomiting or chronic illness was reported. She was initially managed for cervical spine injury and referred to our centre. At presentation, Glasgow Coma Scale (GCS) was as follows: eye response,2; verbal response 2; and motor response 1, with paradoxical breathing, pooling of oral secretions, hypotonia in all limbs, and absent reflexes. Pupils were normal. Due to the altered sensorium of the child, sensory loss could not be evaluated.

Given the fall history and focal neck symptoms, cervical trauma was initially suspected. The absence of bite history and local signs added to the diagnostic dilemma. Empirical management was initiated with cervical spine stabilization, tetanus toxoid, and 10 vials of anti-snake venom. The child was intubated under manual in-line stabilisation and ventilated. The 20-minute whole-blood clotting test was negative. One hour later, she developed hypotension with blood pressure of 74/50 millimetres of mercury (mm Hg) managed with fluid bolus. Central venous access was obtained, and blood pressure stabilized. The GCS had slight improvement. Ventilation required high pressures, raising concern for lung pathology, but auscultation was clear.

Six hours later, reflexes became elicitable although plantar reflexes remained mute, suggesting neuromuscular dysfunction. A second dose of 10 vials of anti-snake venom was given, along with atropine and neostigmine. After another hour, she again developed hypotension with cold peripheries. Noradrenaline was started. Hydrocortisone and chlorpheniramine were given to manage suspected anaphylaxis related to anti-snake venom. Fraction of inspired oxygen was increased to 100% due to desaturation (oxygen saturation, 86%). Point-of-care ultrasound showed B-lines suggesting pulmonary edema. Intravenous furosemide was given, and oxygenation improved.

Computed tomography showed no injury. Hyperthermia of greater than 105 °F was managed with antipyretics and fluids. Intravenous antibiotics were added empirically. Arterial blood gas revealed respiratory acidosis pH of 7.20 (reference range 7.35–7.45); partial pressure of carbon dioxide 62.4 mm Hg (35–45 mm Hg); and lactate 1.23 millimoles per liter (mmol/L) (0.5–1.6 mmol/L). Inotropes were escalated. Differential diagnoses included the following: neurotoxic envenomation (possible snakebite); meningitis; brainstem dysfunction; Guillain-Barré syndrome; pulmonary edema/anaphylaxis related to anti-snake venom; and cervical spine injury.

*CPC-EM Capsule*

What do we already know about this clinical entity?

*Neurotoxic snakebite causes descending paralysis and respiratory failure, often with minimal local signs. Atypical or non specific presentations causes delayed diagnosis*.

What makes this presentation of disease reportable?

*Unilateral neck pain with restricted neck movement and a misleading trauma history mimicked cervical injury, delaying diagnosis of neurotoxic envenomation in a child without bite history*.

What is the major learning point?

*Acute flaccid paralysis despite imaging status in endemic areas should prompt early consideration of neurotoxic snakebite, even without bite history or bite marks*.

How might this improve emergency medicine practice?

*Early suspicion and empiric antivenom in atypical paralysis can prevent fatal delays caused by anchoring bias toward trauma diagnoses*.

More than 24 hours after presentation, the patient developed pulseless ventricular tachycardia. Resuscitation was unsuccessful, and she was declared dead. Due to the sudden unexplained death, medicolegal autopsy was performed. During autopsy, external examination revealed two scabbed puncture wounds over the right lateral malleolus, suspected to be the bite site. Internally, lungs were congested with haemorrhagic areas and frothy secretions with histopathology confirming diffuse pulmonary edema and pneumonitis. Histopathology also showed mild cerebral edema and neuronal swelling.

Skin tissue from the bite site along with control from opposite leg was preserved and sent to the forensic science laboratory. The lab report confirmed the presence of snake venom using immunodiffusion with polyvalent anti-snake venom antibodies. The final cause of death was certified as neurotoxic snakebite leading to respiratory failure and cardiac arrest.

## DISCUSSION

Neurotoxic snakebite, particularly from cobras and kraits, can present with minimal local signs and vague systemic symptoms.^9^ In endemic regions, atypical presentations can delay appropriate treatment, especially when misleading histories such as trauma are present. In the case of our patient, anchoring bias and limited history delayed recognition. Initially, cervical spine trauma was suspected based on the history of fall, neck pain, and progressive weakness. Neck symptoms, hypotonia, areflexia, and later respiratory distress suggested a high cervical lesion. Cranial nerve signs, including ptosis, were misinterpreted as Horner syndrome.

There were no systemic signs of infection, no gastrointestinal or seizure symptoms, and no local signs of envenomation. The whole-blood clotting test was negative. Classic signs such as ptosis and ophthalmoplegia were absent at onset. However, signs such as preserved pupillary reflexes, generalized areflexia, pooling of secretions, and symmetrical flaccid paralysis were more consistent with neuroparalysis. Reflex improvement following administration of anti-snake venom and neostigmine supported reversible neuromuscular blockade.

This is likely the first reported case of neurotoxic envenomation with isolated neck pain progressing to paralysis. While neck weakness has been documented in other case reports, localized unilateral neck pain as the first symptom of envenomation has not been previously reported. While the presenting clinical picture supported cervical trauma, the absence of sensory loss and the presence of symmetrical descending weakness suggested neurotoxic envenomation. Such subtle clues, even without bite history, should raise suspicion—especially in endemic settings.

Prior literature confirms that elapid venom acts at the neuromuscular junction, with venom of the krait (containing β-bungarotoxin) causing presynaptic acetylcholine blockade.^10^ The toxin leads to flaccid paralysis that may not respond to neostigmine if treatment is delayed. Kraits are nocturnal, and victims are often bitten during sleep, typically without local swelling or visible marks. Cobra venom, by contrast, is less potent and produces deeper bites, more prominent local signs, and earlier cranial nerve involvement.

This case emphasizes the need to suspect snakebite in children with acute flaccid paralysis—even with misleading histories. Antivenom should be administered early based on clinical judgment rather than awaiting confirmation. In this case, the anti-snake venom was given empirically 12 hours post-bite, but delayed diagnosis likely contributed to the fatal outcome. Delay in administration of anti-snake venom worsens outcomes. Antivenom is most effective in neurotoxic envenomation before full-blown paralysis sets in.^11^ Supportive care, including mechanical ventilation, neostigmine, and atropine, was appropriately attempted. Pulmonary edema after anti-snake venom use, whether due to toxin-mediated capillary leak or anaphylaxis, has been previously described.^12^

Forensically, histopathology is non-specific (eg, pulmonary oedema).^13^ However, postmortem venom detection from skin near the suspected bite-site provides reliable confirmation, even without visible marks or history. Such confirmation is critical for legal certification and family compensation in India, where snakebite deaths are deemed “unnatural.”^14^, ^15^ Finally, this case reinforces the World Health Organisation’s call for improved snakebite prevention and response strategies: public education; elevated sleeping,;early health-seeking behaviour; and rapid access to anti-snake venom and critical care services.

Delayed presentation is common in rural India due to limited infrastructure and delayed referrals. In this case, critical time was lost due to misdiagnosis at the peripheral centre. Despite resuscitation, the late presentation and delayed treatment worsened the patient’s outcome. From a legal standpoint, snakebite deaths require inquest and autopsy. Diagnostic ambiguity can delay or deny family compensation. Thus, accurate confirmation through methods such as venom testing of skin from the bite-site is vital.

Key implications in this case study include the following: snakebites masquerading as spinal trauma; clinician awareness in endemic areas; forensic lab’s vital role in confirming envenomation in ambiguous or delayed cases; standard protocols for early sample collection; availability of point-of-care venom-detection tools; public health education on early recognition; wearing protective clothing; and seeking immediate care.

## CONCLUSION

Neurotoxic snakebite can mimic other emergencies, such as cervical spine injury, particularly when the history is ambiguous. Clinicians in endemic regions must maintain high suspicion for snakebite in any child presenting with acute flaccid paralysis, cranial nerve involvement, and normal imaging. Awareness of atypical presentations, as in this case, is vital to improve outcomes in endemic areas. This case also demonstrates the importance of forensic infrastructure to ensure accurate death certification, epidemiological reporting, and timely compensation for families.
